# Potential Mechanisms of Muscle Mitochondrial Dysfunction in Aging and Obesity and Cellular Consequences

**DOI:** 10.3390/ijms10010306

**Published:** 2009-01-13

**Authors:** Emilie Chanséaume, Béatrice Morio

**Affiliations:** 1 INRA, UMR1019 Nutrition Humaine, F-63120 Saint Genès Champanelle, France. E-Mail: emilie@nutrifizz.fr; 2 Université Clermont 1, UFR Médecine, UMR1019 Nutrition Humaine, F-63000 Clermont-Ferrand, France

**Keywords:** Mitochondrial dysfunction, skeletal muscle, metabolic disorders, obesity, insulin resistance, type 2 diabetes

## Abstract

Mitochondria play a key role in the energy metabolism in skeletal muscle. A new concept has emerged suggesting that impaired mitochondrial oxidative capacity in skeletal muscle may be the underlying defect that causes insulin resistance. According to current knowledge, the causes and the underlying molecular mechanisms at the origin of decreased mitochondrial oxidative capacity in skeletal muscle still remain to be elucidated. The present review focuses on recent data investigating these issues in the area of metabolic disorders and describes the potential causes, mechanisms and consequences of mitochondrial dysfunction in the skeletal muscle.

## 1. Introduction

Over the past decade, the list of publications suggesting an involvement of mitochondrial oxidative capacity in skeletal muscle in the aetiology of metabolic disorders such as obesity, insulin resistance or type 2 diabetes, has been growing steadily. By considering that lifestyle and physical activity, in addition to age, gender and genetic background, influence mitochondrial oxidative capacity in human muscle, it is clear that the understanding of the causes at the origin of oxidative phosphorylation (OXPHOS) activity impairment is far from being accomplished. In this context, the purpose of this review is to highlight recent knowledge regarding the potential causes, mechanisms and cellular consequences of muscle mitochondrial dysfunction.

## 2. Transcriptional regulation of muscle mitochondrial oxidative capacity

**Figure 1.**
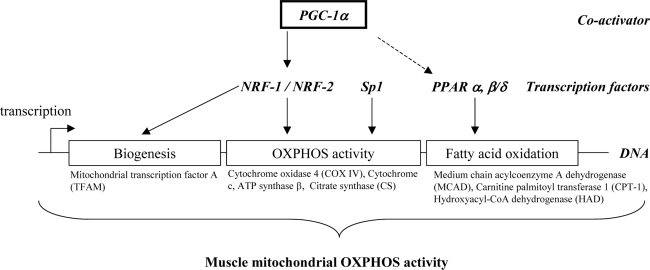
Major transcription factors involved in the regulation of muscle mitochondrial oxidative and phosphorylation (OXPHOS) activity. Non exhaustive key genes, whose expression is regulated by the transcription factors, are given for example.

Muscle oxidative capacity is mainly determined by the mitochondrial density that depends on mitochondrial biogenesis (i.e. the cellular processes involved in the synthesis of the organelles), and the mitochondrial oxidative capacity, which relies on the oxidative enzyme content and activity. A number of transcriptional modulators have been implicated in the regulation of muscle mitochondrial biogenesis and OXPHOS activity. They include PPAR gamma coactivator 1 alpha (PGC-1α), in cooperation with several factors such as the peroxisome proliferator-activated receptors (PPAR), the nuclear respiratory factors 1 and 2 (NRF-1 and NRF-2) [1–9], or the specificity protein 1 (Sp1), an ubiquitous transcription factor known to regulate the constitutive expression of oxidative OXPHOS genes [10]. Of note, Sp1 can function as both a positive (e.g. cytochrome c1 and mitochondrial transcription factor A, TFAM) and a negative (e.g. adenine nucleotide translocator 2 and F1-ATPase beta subunit) regulator of transcription [11]. PGC-1α is a master modulator of gene expression in skeletal muscle [12]. It was found to drive the formation of oxidative type I fibres and to activate the expression of genes involved in mitochondrial oxidative capacity, through associated changes in the expression of NRF dependent genes [13]. These combined data have therefore suggested that decreased PGC-1α gene expression could be one of the primary contributors to decreased mitochondrial oxidative capacity. However, PPARs are also good candidates. When bound to their ligand (e.g. fatty acids for PPARα), PPARs form a heterodimeric complex with the retinoid X receptor (RXR) to regulate gene transcription involved in fatty acid metabolism. PGC-1α is also known to enhance the activity of the isoforms PPARα and PPARβ/δ in skeletal muscle, which may result in the enhanced expression of genes involved in mitochondrial fatty acid oxidation [14]. Major transcriptional modulators involved in the regulation of mitochondrial activity in skeletal muscle are illustrated in Figure 1 (see also [14–16] for reviews). All factors mentioned above co-regulate the transcriptional activity of a variety of genes involved in mitochondrial biogenesis, OXPHOS activity and fatty acid oxidation. For example, in muscle cells, overexpression of PGC-1α was shown to induce the gene expression of NRF-1, NRF-2, TFAM and mitochondrial-encoded cytochrome c oxidase (COX) subunits [15]. Likewise, muscle–specific overexpression of PPARβ/δ in mice was shown to increase oxidative enzyme activities such as citrate synthase or b-hydroxyacyl-CoA dehydrogenase, and to enhance expression of genes implicated in fatty acid catabolism [17].

## 3. Potential causes of impaired mitochondrial oxidative capacity

Emerging data support the hypothesis that in the particular case of young healthy insulin resistant offspring of parents with type 2 diabetes, alteration in mitochondrial activity would be partly inherited [18–21]. In this review, we will only focus on the potential causes of acquired impairment in muscle mitochondrial OXPHOS activity.

### 3.1. Aging

Most studies conducted in animals agree and evidence a decrease in mitochondrial oxidative capacity associated with aging [22, 23]. By contrast, data for humans are still controversial [24–29], although several studies have identified some major pathways altered with aging: 1) decreased maximal activity of key mitochondrial oxidative enzymes (e.g. cytochrome c oxidase or citrate synthase) [30, 31], 2) decreased mitochondrial ATP production rates [19,32], 3) decreased protein content in ATP synthase subunits β [33], and 4) decreased mitochondrial density [30, 34]. In addition, several studies have suggested the involvement of alterations in mitochondrial protein synthesis rate in the age-related decrease in mitochondrial oxidative capacity of skeletal muscle [35, 36]. Indeed, a selective decline in the fractional synthesis rate (FSR) of muscle mitochondrial proteins [37, 39] and a decreased stimulation of mitochondrial protein synthesis in response to insulin [39] have been described in elderly individuals compared to young adults. It remains, however, controversial whether the reduction in mitochondrial oxidative capacity is due to a consequence of aging *per se* or to environmental or lifestyle variables. Indeed, increased sedentary lifestyle and insulin resistance characterizes aging and both situations may contribute to age-related muscle mitochondrial dysfunction.

### 3.2. Insulin resistance

Several studies conducted in a miniature pig model [38], but also in humans [40, 41], have pointed out that insulin infusion acutely and specifically stimulates muscle whole mitochondrial protein synthesis. In addition, muscle mitochondrial ATP production, cytochrome c oxidase (COX) and citrate synthase enzyme activities in association with mRNA levels from both mitochondrial [NADH dehydrogenase subunit IV] and nuclear [cytochrome c oxidase (COX) subunit IV] genes encoding mitochondrial proteins were increased by insulin in skeletal muscle of healthy adults [41]. The study performed in insulin resistant patients with type 2 diabetes demonstrated a diminished stimulation of muscle mitochondrial ATP production by insulin [41]. Similarly, Petersen *et al*. reported decreased insulin-stimulated ATP synthesis and phosphate transport in muscle of insulin-resistant offspring of type 2 diabetic parents [20]. It was therefore suggested that impaired insulin action in skeletal muscle could contribute to the decline in mitochondrial OXPHOS activity [42]. However, in contrast to these results, Southgate et *al*. found using cultured human skeletal myotubes, that insulin acutely decreases the expression of genes involved in oxidative metabolism in healthy but not in insulin resistant muscle [43]. The blunted inhibitory effect of insulin was due to a decrease in the phosphorylation and nuclear exclusion of forkhead box class-O1 (FoxO1) that regulates PGC-1α transcription, secondary to reduced Akt activity. Hence, the hypothesis pointing insulin as a cause of alterations in mitochondrial OXPHOS activity deserves further investigation.

### 3.3. Sedentary behavior

Physical activity and sedentary behaviour play an important role in the regulation of muscle mitochondrial oxidative capacity. In this respect, it has been demonstrated that sedentary behaviour has deleterious consequences on muscle mitochondrial oxidative capacity [29, 44]. Furthermore, the inverse relationship between aging and mitochondrial respiration is no longer valid when the young and the older subjects are matched for a similar level of physical activity [24,29]. Indeed, Rimbert *et al*. used a cross-sectional protocol based on a Latin square design in association with a rigorous selection on subjects’ lifestyle to precisely determine the effects of age *per se* on muscle fat oxidative capacity [29]. The main conclusion arising from the experiment was that muscle mitochondrial OXPHOS activity and the resultant muscle fat oxidative capacity were not primarily impaired by age but by physical inactivity. The importance of physical activity in the prevention of muscle deconditioning and metabolic disorders in young and elderly people should be therefore taken into account.

### 3.4. Nutrition quality and diet-induced obesity

Nutritional state (e.g. obesity) and nutrients may contribute to skeletal muscle dysfunction. It is now recognized that nutrients have the ability to interact with transcription factors and contribute to biological processes. In this context, new disciplines have recently emerged in the field of nutrition i.e. nutrigenomics and nutrigenetics. Nutrigenomics examines the impact of dietary habits (nutrients provided by diet) on the genome. Nutrigenetics investigates the effect of genetic variation on the interaction between diet and disease. Humans considerably vary in their individual responses to diet, and such approaches can help to examine the impact of nutrition on skeletal muscle function.

Limited but significant data support the concept that nutrition, both quantitatively and qualitatively, may be directly responsible for changes in muscle mitochondrial function. Results have evidenced that in rats, high-fat or high-sucrose intake, and more importantly overfeeding (which effects have to be related to those observed with obesity), are major factors associated with decreased muscle mitochondrial OXPHOS activity, but in a muscle-specific fashion [45]. The mitochondrial OXPHOS activity within the oxidative muscle *soleus*, resistant to fatigue and dependent on mitochondrial activity for ATP production, were more affected than within the glycolytic muscle *tibialis anterior*. The main changes included a reduction in the respiratory chain activity with a concomitant decrease in mitochondrial ATP production [45]. Decreased mitochondrial respiration rates [46] and reduced expression of genes involved in mitochondrial oxidative capacity [47] have been reported in diet-induced obese rats. Judging by the available data, excess energy intake and obesity, can be directly associated with alterations in muscle mitochondrial activity. Yet, the role of obesity and in particular the type of obesity (i.e. android or gynoid) in the induction of muscle mitochondrial disturbances is an important question that needs to be addressed.

## 4. Potential mechanisms: intrinsic factors

As previously described, the transcriptional coactivator PGC-1α is a potent stimulator of mitochondrial biogenesis and OXPHOS activity (c.f. part 1). A decrease in the expression of mitochondrial genes associated with a concomitant reduction in NRF-1 and PGC-1α gene expression has been reported in the skeletal muscle of insulin resistant and diabetic patients [7, 48]. However the intrinsic factors responsible for depressed NRF-1 and PGC-1α gene expression remain to be fully elucidated. There are multiple processes by which environmental or physiologic factors might play a critical role in the control of mitochondrial biogenesis and function. Among these mechanisms, we selected lipotoxicity, inflammation and glucotoxicity.

First, *lipotoxicity* is the overall damage caused to tissues secondary to prolonged exposure to high levels of plasma non-esterified free fatty acids (NEFA). Excess NEFA and the accumulation of intramyocellular lipid metabolites (e.g., diacylglycerol, fatty acid acylcoA or ceramides) consecutive to increased NEFA availability, have been demonstrated to trigger insulin resistance [49, 50] but may also disturb mitochondrial activity. Recent findings on cultured skeletal muscle cells have brought evidence that fatty acids play a significant role in the regulation of muscle oxidative metabolism. First, a study on human myotubes reported that PGC-1α gene expression was increased two- to three-fold by unsaturated fatty acids but was unchanged with saturated fatty acids [51]. Mitochondrial activity was concomitantly enhanced by unsaturated fatty acids, but was impaired with the saturated fatty acid stearate [51]. Second, data obtained on C2C12 showed that the saturated fatty acid palmitate, by contrast to the monounsaturated fatty acid oleate, reduces PGC-1α gene expression through a mechanism involving mitogen-activated protein kinase (MAPK), extracellular signal-related kinase [52] and NF-κB activation [53]. Another study showed that saturated fatty acids decrease PGC-1α and mitochondrial gene expression and function via p38 MAPK-dependent transcriptional pathways [54]. Interestingly, Benton *et al*. [55] showed in two animal models in which muscle fatty acid accumulation was either increased (Zucker obese rats) or decreased (FAT/CD36 null mice) that PGC-1α protein expression was inversely correlated to the cellular ability to uptake and store lipids. Similarly, adult rats fed a high-fat diet for two weeks showed lower muscle mitochondrial respiration rates than low-fat fed rats [46].

An original study recently showed that resveratrol, a phytoalexin found in particular in the skin of red grapes, can reverse the deleterious effects of high-fat diet on muscle mitochondrial function in mice [56]. Resveratrol treatment greatly enhanced mitochondrial oxidative capacity, by induction of genes involved in oxidative phosphorylation and mitochondrial biogenesis. These adaptations were principally explained by a resveratrol-mediated activation of the protein deacetylase Sirt1, the result being a decrease in PGC-1α acetylation and an increase in PGC-1α activity [56]. Additional evidence came from research performed in humans. A study conducted in young men has demonstrated that 3-day high-fat diet decreases the expression of genes involved in mitochondrial oxidative capacity in skeletal muscle [8]. To strengthen the lipotoxic theory, infusion of intralipid for 48 hours in healthy humans decreases the muscle expression of both PGC-1α and several genes involved in oxidative phosphorylation [57]. Thus, consistent data obtained on various models, from cell to human, show that intramuscular lipid sensing may be involved in regulating the muscle PGC-1α expression and activity, and consequently muscle mitochondrial oxidative capacity, although further investigations are required to determine quality, dose and time-dependent effects of fatty acids on muscle mitochondrial OXPHOS activity. This single mechanism could be suitable to explain most of the muscle mitochondrial adaptations with metabolic disorders such as obesity and type 2 diabetes. However, additional complex interactions and pathways are likely to occur.

In that respect, a number of *adipocyte-derived factors* may be responsible for the reduced mitochondrial oxidative capacity. Adipose tissue makes up ~ 15–25% of body mass in men and women with normal values of body mass index (BMI = 18–25 kg/m^2^) but can vary from 4–10% in athletes to 50% in obese patients (BMI > 30 kg/m^2^). Adipose tissue cells comprise adipocytes and non-adipose cells that constitute the stroma-vascular fraction, mainly endothelial cells, leucocytes, monocytes and macrophages. Recent studies support the hypothesis that obesity is associated with a state of chronic low-grade inflammation [56–61]. Tumor necrosis alpha (TNF-α) and interleukin 6 (IL-6), pro-inflammatory cytokines produced mainly by adipocytes and macrophages but also by muscle cells, are up-regulated in obesity [58–60]. Recently, it has been shown that TNF-α might positively autoregulate its own synthesis in adipose tissue [61], which might contribute to the maintenance of the elevated TNF-α observed in obesity [62, 63]. One should also keep in mind that the saturated fatty acid palmitate enhances TNF-α expression in skeletal muscle [64]. TNF-α signaling through TNF receptor has been implicated in the pathogenesis of insulin resistance [58–60, 62–63]. In vitro and in vivo data have shown that it suppresses AMPK activity via transcriptional upregulation of protein phosphatase 2C (PP2C). This in turn reduces the phosphorylation of the enzyme acetyl-CoA carboxylase, suppressing fatty-acid oxidation, increasing intramuscular diacylglycerol accumulation, and causing insulin resistance in skeletal muscle [65]. Importantly, TNF-α has been shown to increase the expression of the inducible isoform of the nitric oxide synthase (iNOS) [66] and to downregulate that of the endothelial isoform (eNOS) [67]. Decreased expression of the neuronal isoform (nNOS) has been also reported in skeletal muscle of streptozotocin-induced diabetic rats [68]. These enzymes catalyze the biosynthesis of NO (a short-lived highly diffusible hydrophobic free radical) from L-arginine and molecular oxygen utilizing NADPH as an electron donor and heme, FMN, FAD and tetrahydrobiopterin (H4B) as cofactors [69]. It is now demonstrated that NO generated by eNOS increases mitochondrial biogenesis, oxidative metabolism and ATP levels in several cell types, including muscle [70, 71]. Hickner *et al*. [72] have shown that in young women skeletal muscle eNOS protein content and activity are inversely related to body fat percentage. In addition recent evidences have demonstrated that eNOS expression and mitochondrial biogenesis are downregulated in adipose and muscle tissues of genetically and diet-induced obese mice and rats whereas iNOS is upregulated [67]. This process has been shown to be partly mediated by cGMP, resulting from NO-dependent activation of “soluble” guanylate cyclase, and involves the increased expression of PGC-1α, NRF-1 and TFAM [70]. Thus, in vitro and in vivo data support that TNFα may be involved in regulating muscle PGC-1a expression, and consequently muscle mitochondrial oxidative capacity.

Regarding IL-6, Al-Khalili *et al*. have recently established on culture cells from skeletal muscle that IL-6 regulates muscle substrate utilization, enhancing glycogen storage and lipid oxidation [72]. Yet based on the current data, it can be assumed that IL-6 exerts different effects according to the physiological situation, i.e. in response to exercise [73] or during low-grade inflammation in obesity [74, 75]. By contrast to the pro-inflammatory cytokines TNF-α and IL-6, IL-15 is a cytokine highly expressed in skeletal muscle which induces fatty acid oxidation [73] and facilitates glucose metabolism [74]. Furthermore, leptin and adiponectin are anti-inflammatory hormones exclusively produced by adipocytes [76, 77]. Leptin is an adipocytokine produced proportionally to adipose tissue size [78], initially described for its action in brain regions to reduce food intake. Adiponectin, which is present at a high concentration in the plasma, is downregulated with obesity [58]. Leptin and adiponectin have been shown to activate muscle fatty acid oxidation and this action appears to be mediated by AMP-activated protein kinase (AMPK) activation that triggers stimulation of mitochondrial function and biogenesis [79,80]. Indeed, activation of AMPK enhances PGC-1α gene expression [75] and stimulates PPARα [80, 82]. The balance between pro-inflammatory factors and adipocytokines may be therefore a connective link between adipose tissue mass and function, and metabolic disorders in skeletal muscle [81].

Finally, *glucotoxicity* is commonly defined by the overall damage caused to tissues, secondary to prolonged exposure to elevated plasma glucose concentration. The degree of mitochondrial failure has been correlated with the duration of diabetes. Complexes I, III and IV of the electron transport chain have been shown to be the main mitochondrial targets of hyperglycaemia-induced injury [76]. The presence of chronic hyperglycaemia can cause structural alterations of proteins through the Maillard reaction, and can lead to oxidative stress, a state of imbalance between the production of reactive oxygen species (ROS) and antioxidant defences, and consequently to cellular oxidative damage [77, 78]. In that respect, recent evidences have demonstrated that oxidative stress in skeletal muscle is probably one of the major determinants of the mitochondrial alterations in obesity and type 2 diabetes [79]. This is supported by in vivo and in vitro data showing that 1) an increase in muscle ROS production occurs specifically after hyperglycaemia and hyperlipidemia have appeared in high fat fed mice; 2) in this model, normalization of glycaemia by insulin or phlorizin and treatment with an antioxidant (*N*-acetylcysteine) decreases muscle ROS production and restores mitochondrial integrity; 3) incubation of cultured muscle cells with high glucose or lipid concentrations induces ROS production and alters mitochondrial density and functions; 4) these effects are blocked by an antioxidant treatment. Enhanced mitochondrial ROS production has been also shown to activate the redox-sensitive transcription factor NF-κB [80], which has been associated with PGC-1α downregulation in C2C12 skeletal muscle cells [53]. In addition, ROS overproduction is likely to enhance several metabolic pathways such as the hexosamine biosynthesis pathway (HBP) [81]. HBP is a nutrient-sensing pathway that has been implicated in the development of insulin resistance [81]. Obici et al. have reported that HBP activation in response to short-term overfeeding is accompanied by an inhibition of the expression of genes (e.g. malate dehydrogenase, acyl-CoA dehydrogenase, propionyl-CoA carboxylase, subunits of complexes I, III, IV and V, adenine nucleotide translocator 2, mitochondrial 2-oxoglutarate/malate carrier protein) involved in mitochondrial oxidative capacity within skeletal muscle [47]. The molecular mechanisms may in part relate to the control of Sp1 activity via *O*-linked *N*-acetylglucosamine (O-GlcNAc) modification [11].

## 5. Cellular consequences of impaired mitochondrial oxidative capacity

Skeletal muscle is highly dependent on mitochondrial oxidative phosphorylation for ATP production, the major energy source for prolonged muscle activity. It is obvious that muscle mitochondrial density and oxidative capacity adapt to muscle energy demand, and therefore decrease with physical inactivity. However, one can question why the muscle mitochondrial oxidative capacity should decrease in a situation of excess nutrient availability (excess intakes of energy, lipid or glucose, obesity). Can one consider this adaptation as a cell suicide? Do we reach the limits of the cellular adaptation? Or are they any beneficial consequences of such an adaptation? Interestingly, Obici *et al*. [47] suggested that, in line with the thrifty genotype hypothesis, decreased mitochondrial oxidative capacity in the presence of enhanced nutrient availability in skeletal muscle may have conferred a selective survival advantage by favouring the storage of excess nutrients as fat during periods of sporadic food availability. Far from willing to propose an answer, we will briefly review the potential cellular consequences of decreased muscle mitochondrial OXPHOS activity.

### 5.1. Aerobic capacity

Aerobic capacity is quantified through VO_2_ max, the maximal oxygen uptake capacity. Limiting factors for VO_2_ max involved principally the cardiorespiratory system. But endurance performance is also strongly related to mitochondrial oxidative capacity [82]. The latter, especially in sedentary individuals, has been proposed to play a critical role in limiting the oxidative metabolism of skeletal muscle, to a larger extent than oxygen supply [83]. Intolerance to prolonged exercise and early fatigability are common features associated with defects in muscle oxidative capacity [84, 85]. Abnormal response to exercise has notably been associated with a reduced oxidative enzyme activity of complexes III [86] and IV [87] of the mitochondrial respiratory chain.

### 5.2. Oxidative stress

Mitochondria are critical organelles involved in the generation of ROS. The normal functioning of the mitochondrial respiratory chain continually produces ROS, principally superoxide anion and nitric oxide. ROS have a very short half-life but can rapidly react with DNA, proteins and lipids causing damages to all cell components, including an increased mutation rate for DNA or an increased formation of oxidized proteins and lipids. The mitochondrial membranes and DNA are particularly vulnerable to oxidative stress but all cellular structures are concerned. Oxidation of proteins may alter their structure and function either by loss of catalytic enzyme activity and structural integrity or by interruption of regulatory pathways. Fatty acids are also particularly prone to oxidative damage, resulting in the formation of lipid peroxides. Russel et al. found that skeletal muscle of obese insulin-resistant subjects contained a higher amount of intramyocellular lipids, and a higher degree of lipid peroxidation [88]. Alterations of membrane components also lead to cell dysfunction and even to cell death. For instance, the oxidation of mitochondrial cardiolipins is a key factor in the initiation of cell apoptosis [89]. Although consequences of increased oxidative stress have been clearly identified [90–93], the involvement of mitochondrial dysfunction in increased ROS production associated with metabolic disorders is still under debate. In that respect, Chanseaume *et al*. have shown that adaptations in mitochondrial OXPHOS activity in rats receiving high-energy diets were associated with a reduction in muscle mitochondrial superoxide anion production [45]. Consistent with comments from Obici *et al*. [47], decreasing respiratory chain activity in skeletal muscle may not only be considered detrimental to ATP synthesis but may also be responsible for reduced ROS production. This could be related to decreased levels of mitochondrial oxidative protein damage observed in the muscle of diabetic Sprague-Dawley rats [94].

### 5.3. Metabolic flexibility

Metabolic flexibility describes the ability of muscle to switch between glucose and fatty acids as oxidative energy source depending on metabolic conditions and energy demand. In the healthy state, muscle may switch from fatty acid oxidation under fasting conditions to increased glucose oxidation in the postprandial state. Such capacity to switch between fuels is strongly reduced in obese and diabetic individuals as a consequence of impaired oxidative capacity or/and insulin resistance [95]. This observation supports the hypothesis that alterations in skeletal muscle mitochondrial activity might lead to abnormalities in fuel selection and partitioning, and participate to metabolic inflexibility [96].

### 5.4. Intracellular lipid content and insulin sensitivity

Although controversial [97, 98], a recent concept has proposed that any impairment of mitochondrial function might predispose to intramyocellular lipid (IMCL) accumulation (fatty acids and/or lipid metabolites). Some evidence has been brought by Benton *et al*. using animal models characterized by high or low muscle ability to uptake and store fatty acids [56]. PGC-1α protein expression was inversely correlated to the cellular IMCL synthesis rate in the presence of fatty acids. Furthermore, increased triglyceride storage has been observed in parallel to decreased fatty acid oxidative capacity in skeletal muscle of obese and diabetic subjects [99–101].

It is now well established that mitochondrial oxidative capacity is linearly correlated to insulin sensitivity within skeletal muscle [29]. In addition, a decrease in mitochondrial content and function has been described in the skeletal muscle of obese, insulin resistant and type 2 diabetic patients compared to healthy individuals [7, 19–20, 30–31, 52, 101–104]. Hence in the past few years, muscle mitochondrial dysfunction has been suggested as the leading cause for impaired insulin sensitivity [21]. The close association between mitochondrial oxidative capacity and insulin sensitivity possibly involves alterations in intracellular trafficking of fat metabolites [103, 105]. Recent studies using *in vivo* nuclear magnetic resonance (NMR) spectroscopy have evidenced an inverse relationship between insulin sensitivity and ICML content [106–108]. The molecular mechanism underlying defective insulin-stimulated glucose transport activity may be attributed to an accumulation of intramyocellular lipid metabolites such as ceramides, fatty acyl CoAs and diacylglycerol which could potentially disrupt the insulin signalling pathway through Ser/Thr phosphorylation of insulin receptor substrate [48]. A convincing demonstration has been brought by Petersen *et al*. [27] who compared IMCL content and mitochondrial function in healthy young and elderly individuals using NMR spectroscopy. Data demonstrated that elderly individuals showed significantly higher ICML, but reduced muscle mitochondrial ATP synthesis and higher plasma insulin concentration during oral glucose tolerance test compared to young adults [27]. But while several authors support this hypothesis using transversal studies, results from recent work have questioned this concept. In a chronological model of diet-induced obesity in rats, it has been demonstrated that insulin resistance can not be attributed to a decrease in mitochondrial oxidative capacity, which appears later, after changes in lipid metabolism and insulin sensitivity have occurred [109]. The main result of the latter experiment was that before being a “victim”, muscle mitochondrial oxidative capacity first positively adjusts to excess energy and contributes to limit the diet-induced metabolic disorders i.e. alteration of lipid metabolism, IMCL accumulation and insulin resistance [109]. Therefore new possibility has emerged that mitochondrial dysfunction is not necessarily the primary cause of IMCL accumulation and insulin resistance within skeletal muscle but that stimulating mitochondrial OXPHOS activity may be of importance in the prevention of these metabolic disorders [2, 117].

## 6. Conclusions

**Figure 2.**
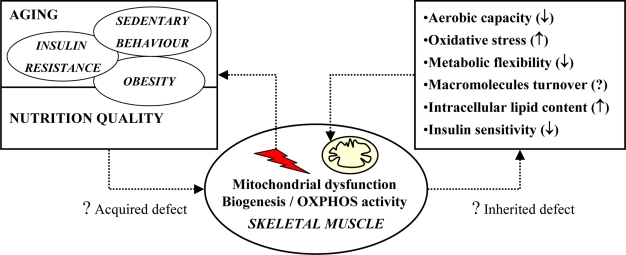
Summary of potential causes and cellular consequences of impaired mitochondrial oxidative and phosphorylation (OXPHOS) activity in skeletal muscle. Physiological factors associated to aging, hormonal changes, lifestyle behavior and diet, may impair muscle mitochondrial biogenesis and OXPHOS activity during lifespan. These alterations, named “acquired defects”, in association with “inherited defects” due to genetic or epigenetic processes, may favor the apparition of metabolic disorders which then hasten a vicious circle that can ultimately lead to pathological states.

According to current knowledge, the underlying molecular mechanisms of muscle mitochondrial dysfunction in metabolic disorders are far from being elucidated. One or a combination of mechanisms involving genetics, disturbances in glucose and lipid homeostasis, oxidative stress and also a state of chronic low-grade inflammation, might be involved in the process leading to these defects. Figure 2 illustrates potential causes and cellular consequences of impaired mitochondrial oxidative capacity in skeletal muscle. Nowadays, understanding the molecular and biochemical defects responsible for muscle mitochondrial dysfunction is of importance to specify the role of mitochondrial dysfunction in the aetiology of metabolic disorders and to define preventive and therapeutic targets for the treatment of these pathologies.
